# A Comparison of the Celiacomesenteric Trunk in the Caribbean with Global Prevalence Calculated by a Systematic Review

**DOI:** 10.1155/2022/1715631

**Published:** 2022-10-11

**Authors:** Shamir O. Cawich, Maurice Fortune, Rahul Deshpande, Michael Gardner, Neil Pearce, Peter Johnson, Vijay Naraynsingh

**Affiliations:** ^1^Port of Spain General Hospital, Port of Spain, Trinidad and Tobago; ^2^Queen Elizabeth the Queen Mother Hospital in Maragate, Part of the East Kent Hospitals University Foundation Trust, Canterbury, UK; ^3^Manchester Royal Infirmary, Oxford Road, Manchester M13 9WL, UK; ^4^University of the West Indies, Mona Campus, Kingston, Jamaica; ^5^Southampton University Hospital NHS Trust, Southampton, UK

## Abstract

**Background:**

Typically, the celiac trunk and superior mesenteric artery branch off separately from the anterior aspect of the abdominal aorta. The celiacomesenteric trunk (CMT) is a rare variant in which those arteries share a common origin. We sought to compare the prevalence of CMT in the Caribbean with the global prevalence as calculated by a systematic review.

**Methods:**

In this study, we evaluated all consecutive patients who had multiphase contrast-enhanced CT scans at two major referral centres in the Caribbean from August 30, 2017, to September 1, 2019. In patients with a CMT, we recorded demographic and anatomic details. We then conducted a systematic literature search and retrieved raw data to calculate the global prevalence (number of individuals with a CMT divided by the sum total of study samples). We compared CMT prevalence in our sample with the global prevalence using Pearson's chi-square and Fisher's exact tests. Statistical significance was considered to be present when the *P* value was <0.05.

**Results:**

From 832 CTs, 665 scans met the inclusion criteria. There were 16 (2.41%) CMTs: 3 (0.45%) classic CMTs, 12 (1.8%) hepato-mesenteric trunks, and 1 (0.15%) hepato-spleno-mesenteric trunk. Forty-two studies reported on CMTs in a total of 74,320 persons. The global CMT prevalence was comparable (3.88%; *P* = 0.054), but the incidence of hepato-mesenteric variants was significantly lower in our sample (1.8% vs. 3.24%; *P* = 0.0352).

**Conclusion:**

There was no difference in the prevalence of a classic CMT in the Caribbean compared to the global prevalence. However, the hepato-mesenteric trunk (incomplete CMT variant) was significantly less prevalent in the Caribbean. Advances in Knowledge: Healthcare professionals performing hepatobiliary interventions must be aware of these differences in order to minimize morbidity during their interventions.

## 1. Introduction

The celiac trunk (CT) and superior mesenteric artery (SMA) branch off separately from the anterior aspect of the abdominal aorta. The celiacomesenteric trunk (CMT) is a rare variant in which those arteries share a common origin from the abdominal aorta [1]. This variant is clinically important because it may impact invasive procedures on the upper abdominal viscera.

Considering the fact that several variations of arterial supply to the upper abdominal viscera have been reported in persons of Caribbean descent [2–5], we sought to document the prevalence of the CMT in the Caribbean study sample. A secondary aim of this study was to determine whether this was different from the global prevalence as calculated by a systematic review of studies across the world.

## 2. Methods

This study was performed over a 24-month period at major referral centres in two countries in the Anglophone Caribbean [6]. Through an initiative from the Caribbean Chapter of the Americas Hepatopancreatobiliary Association, hepatobiliary referral centres were set up in these countries to serve the entire Caribbean population [7]. At these centres, multidisciplinary teams met weekly to review electronic images and plan the management of patients with liver and pancreatic diseases. The local institutional review board granted approval to review all images during these meetings.

All patients had multiphase computed tomography scans by using 64-slice multirow detector scanners. A nonionic contrast medium, Ultravist 300® (iopromide), in a volume of 100 ml, was routinely administered in all studies by using a pressure injector with bolus tracking. We included all scans with an arterial phase that adequately covered the CT, SMA, and IMA territories. Exclusion criteria included duplicated scans, those with incomplete demographic data, inadequate arterial phases, and patients with prior vascular surgery or abdominal interventional radiology procedures.

### 2.1. Definitions

**Figure 1:**
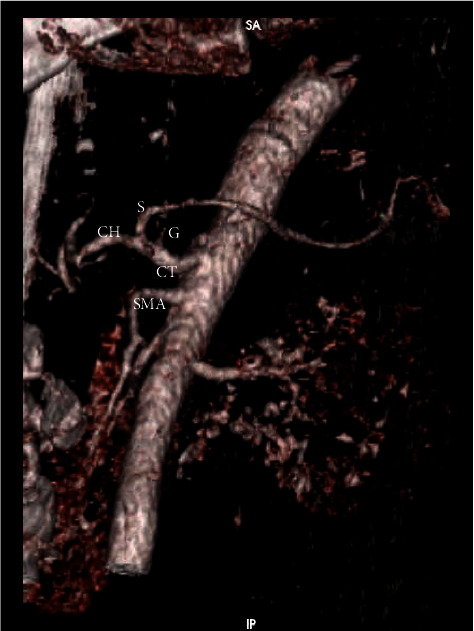
Three-dimensional volume rendering CT image shows normal branching patterns from the abdominal aorta. The celiac trunk (CT) is the most cephalad branch and gives off three branches: the splenic (S), left gastric (G), and common hepatic (CH) arteries. The superior mesenteric artery (SMA) arises at the L1 vertebral level from the aorta.

**Figure 2:**
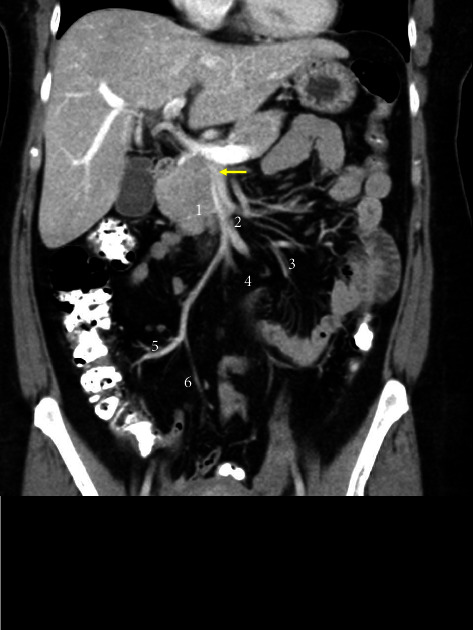
Ramifications of the superior mesenteric artery (arrow) include the inferior pancreaticoduodenal (1), middle colic (2), jejunal (3), ileal (4), right colic (5), and ileocolic arteries (6).

Żytkowski et al. [8] pointed out that there are normal variations in anatomy in all body systems, but there are also classic anatomic descriptions to describe the most common anatomic patterns. We referred to these classic anatomic descriptions of arterial anatomy [1, 9], where three major arteries arise from the anterior aspect of the abdominal aorta to supply the intra-abdominal viscera. The CT scan is most cephalad and branches into the common hepatic, splenic, and left gastric arteries (Figure 1). The SMA gives off the inferior pancreaticoduodenal, middle colic, right colic, ileocolic, ileal, and jejunal arteries (Figure 2). The IMA arises at the third lumbar vertebral level and gives off the left colic, sigmoid, and superior rectal arteries.

Although there are many reported variants, there is no consensus on nomenclature. For the purposes of this study, we defined the CMT as a common arterial channel arising from the abdominal aorta, regardless of its vertebral level, and giving off branches that belong to CT and mesenteric artery territories. We defined two types: complete and incomplete [10–14].

A complete CMT was one in which a single common trunk arose from the aorta and gave origin to all branches of the mesenteric artery and celiac trunk territories. Two complete CMT subtypes were defined: the classic celiacomesenteric trunk (CT and SMA territorial branches) and a celiac-bi-mesenteric trunk (CT, SMA, and IMA territorial branches).

An incomplete CMT was one in which there was a shared origin for at least one arterial branch across the CT and mesenteric artery territories. Nomenclature was based on the branches from the common trunk, regardless of the origin of remaining arteries that did not originate at the shared trunk.  Table 1 summarizes the definitions used in this study for the purposes of classification.

### 2.2. Caribbean Data

Using these definitions, three radiologists independently examined all computed tomography images encountered between August 30, 2017, and September 1, 2019. Image series in which a CMT was thought to be present were selected for detailed re-examination by all three radiologists. These were experienced radiologists who completed specialty training in radiology, each with more than 5 years of experience as consultant radiologists. Patients were only included in the study sample if there was an agreement between all radiologists that a CMT was present. In the event of a disagreement, this was resolved by group discussion and re-examination of images by the three radiologists.

In patients who possessed a CMT, we recorded demographic details and the relevant anatomic details. Data were recorded in a Microsoft Excel sheet. Descriptive analyses were performed by using the SPSS statistical software.

### 2.3. Systematic Literature Review

We then conducted a systematic literature review using medical archiving platforms, including Pubmed, Medline, Google Scholar, and the Cochrane Database of Systematic Reviews. We used the following search terms: “coeliaco-mesenteric trunk,” “coeliaco-bi-mesenteric trunk,” “coeliac axis variants,” “coeliac trunk variants,” “common trunk,”, “gastro-splenic,” “spleno-mesenteric,” “gastro-hepatic,” “spleno-hepatic,” “gastro-colic,” “splenocolic,” “hepato-colic,” and “hepato-mesenteric.” All relevant studies were retrieved, and the data and images were reviewed in detail. We used the raw data from these retrieved studies to calculate the global prevalence of CMTs. Global prevalence was calculated by dividing the number of individuals with a CMT by the sum total of study samples from studies across the globe. The prevalence of the CMT in our study sample was also calculated and compared with the global prevalence. We used Pearson's chi-square and Fisher's exact tests to compare 2 × 2 contingency tables, and statistical significance was considered to be present when the *P* value was <0.05.

## 3. Results

### 3.1. Caribbean Data

A total of 832 CT scans were examined, and 167 scans were excluded from the final study sample for duplications (64), prior intra-abdominal vascular procedures (51), inadequate arterial phase (48), and incomplete demographic data (4). The final study sample comprised 665 scans that met the inclusion criteria. A CMT variant was present in 16 (2.41%) persons in the study sample, as detailed in Table 1. Overall, there was a preponderance of CMT variants in males (10 : 6). All patients were asymptomatic, and there were no clinical sequelae directly attributable to CMT variants in any of these persons.

**Figure 3:**
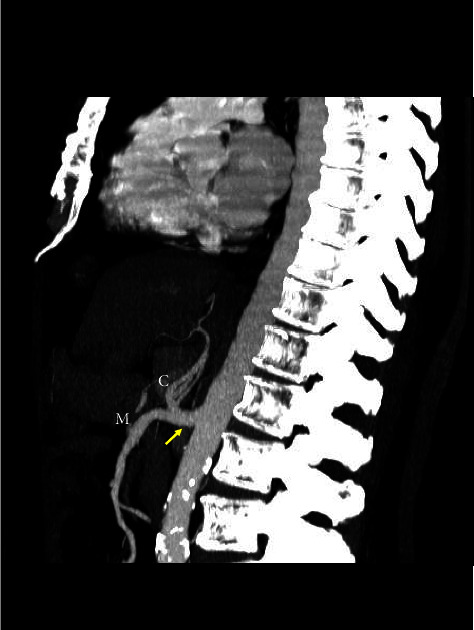
Three-dimensional volume rendering CT image showing a complete celiacomesenteric trunk (CMT) inclusive of mesenteric (M) and celiac (C) ramifications.

**Figure 4:**
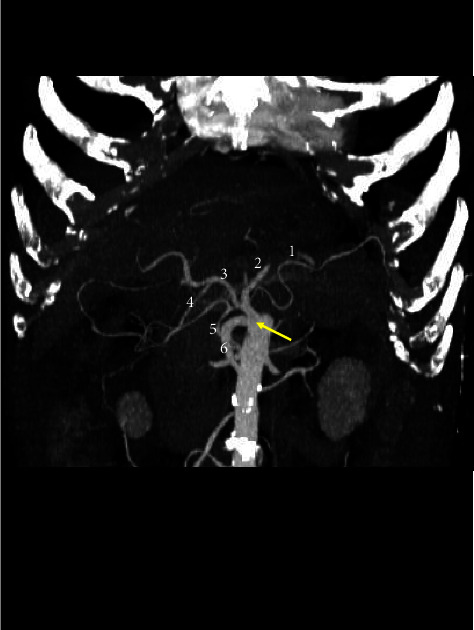
Three-dimensional volume rendering CT image showing a complete CMT (arrow). The celiac branches visible include the splenic (1), left gastric (2), common hepatic (3), and gastroduodenal arteries (4). The mesenteric branches visible include the inferior pancreaticoduodenal (4) and superior mesenteric ramifications (5).

**Figure 5:**
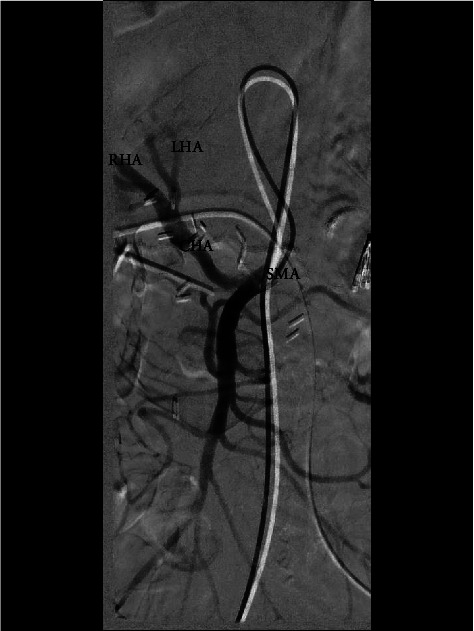
Fluoroscopic images during selective mesenteric angiography showing the common hepatic artery (CHA) arising from the superior mesenteric artery (SMA) and then bifurcating into the left (LHA) and right (RHA) hepatic arteries.

**Figure 6:**
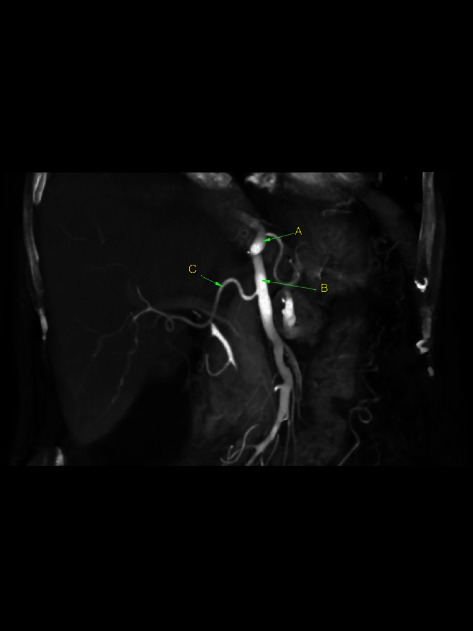
Incomplete CMT: hepato-mesenteric variant showing a replaced right hepatic artery (C) arising from the superior mesenteric artery (B) instead of the celiac trunk (A).

**Figure 7:**
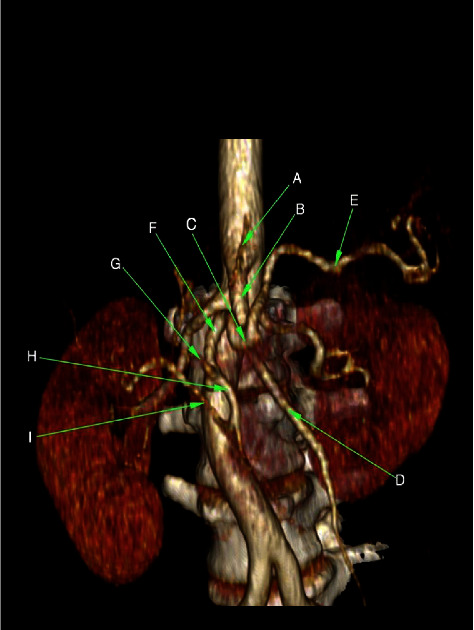
Three-dimensional volume rendering CT image demonstrating a hepato-spleno-mesenteric trunk. *A* = left gastric artery, *B* = celiacomesenteric trunk, *C* = celiac trunk, *D* = superior mesenteric artery, *E* = splenic artery, *F* = common hepatic artery, *G* = left hepatic artery, *H* = gastroduodenal artery, and I = right hepatic artery (reproduced with permission from Johnson PB, et al. Vascular Supply to the Liver: Report of a Rare Arterial Variant. Case Rep Radiology. 2013.969327:1-3. DOI: 10.1155/2013/969327 [2]).

Three (0.45%) males had a complete “classic” CMT (Figures 3 and 4), and there were no persons with a celiac-bi-mesenteric trunk. The most common incomplete CMT variant was a hepato-mesenteric trunk in 12 (1.8%) persons, involving the origin of the common hepatic artery (CHA) in 5 persons (Figure 5), the replaced right hepatic artery (RHA) in 4 persons (Figure 6), and the replaced left hepatic artery (LHA) in 3 persons. The only other incomplete CMT variant was a male with the hepato-spleno-mesenteric trunk (Figure 7).

### 3.2. Systematic Review

In our review of the medical literature, we encountered 42 population-based series that reported the prevalence of CMT variants in a total of 74,320 persons [1, 9–49]. In each study, the raw data were extracted and tabulated for the purpose of data analyses. The raw data and statistical comparisons are presented in Table 2. There was a statistically lower incidence of the hepato-mesenteric variant in this sample (1.8% vs. 3.24%; *P*=0.0352).

## 4. Discussion

The majority of persons in this Caribbean study sample had conventional branching from the abdominal aorta [50–53]. This classic pattern is reported to be present in 44% [13] to 91% [33] of persons in the international literature.

The CMT is recognized as a rare variant. Benjamin Lipshutz is credited with coining the term “*truncus celiaco-mesenterica*” when he described 2 cadavers with a variant where the SMA and CT took a common origin from the aorta [1]. In the subsequent decades, the CMT was documented in case reports [54–66] and larger population-based series [1, 9–49]. These data suggest that CMT occurs in 0.42% [37] to 2.7% [35] of unselected persons across the globe.

The CMT is believed to be an aberration in embryonic development. During embryogenesis, the visceral arteries arise from the primitive dorsal abdominal aorta through four roots (gastric, hepatic, splenic, and superior mesenteric roots) initially joined in a longitudinally-oriented primitive ventral anastomosis [1, 66]. The superior mesenteric root is the dominant arterial root [1] in the primitive ventral anastomosis. Usually, a cleft develops between the third and fourth arterial roots that separate the CT and SMA, respectively [66]. When the primitive cleft does not form, the primitive ventral anastomosis persists, and this gives rise to complete CMT. A partially formed primitive cleft does not separate all the primitive aortic roots and leads to the formation of an incomplete CMT. This was the basis of our classification.

Although many authors have written about CMT, there is no standardized definition in the medical literature. Most authors seem to agree that a CMT exists when there is a “common origin of the SMA and CT,” and it includes the three main CT branches [10, 12–14, 35]. However, some authors used other names to describe the same pattern, such as “gastro-hepato-spleno-mesenteric trunk” [55, 67], “CT arising from SMA” [64], or “persistent anastomotic channel” [27]. Still, others have required extra detail to meet their definition of a CMT. For example, Varma et al. [58] stipulated that the common origin for CT and SMA must *“further divide into hepato-mesenteric and gastro-splenic trunks*” to be defined as a CMT.

To add an additional layer of complexity, some authors include variants with only two main CT branches arising from the common origin in their definition of a CMT [14, 36, 47, 60]. For example, Tang et al. [47] defined the CMT as a “*single common trunk arising from the aorta, and the branches include the SMA and at least two major branches of the CT.”* Whitely et al. [14] also included variants with the SMA plus two major CT branches, defining this as an “incomplete CMT.” Yet, other authors reporting on the CMT ignore variants that include a combination of the SMA plus two CT branches [13, 27, 65]. For example, Bolintineanu et al. [65] reported on the presence of a hepato-spleno-mesenteric trunk that was not considered a CMT and, in fact, discussed CMTs separately in their paper.

Still, other authors include variants where the SMA plus one CT branch has a common origin from the aorta [11, 54, 60], while others do not consider these variants as CMTs [29, 36, 47]. For example, Kornafel et al. [29] excluded a variant in which the common hepatic artery (CT territory) arose from the SMA, instead terming this a “hepato-mesenteric trunk.” Meanwhile, other authors have introduced descriptive terms such as “complete vs. incomplete” CMTs [14, 33, 68] that inconsistently seem to be used interchangeably with “classic vs. variant” CMTs [11].

The wide variation in nomenclature and the multiplicity of classification systems [1, 9, 14, 18, 27, 36, 47, 54, 69] bear testimony to the fact that there is no standardized nomenclature or definition. We based our classification on the basis of embryologic development of the aortic branches, independent of the final ramifications of the CMT branches and/or origins of non-CMT arteries. We avoided numeric classifications that we found confusing and instead attempted to describe CMT ramifications using the combinations of the branch names as determined by their territorial supply. We thought this would allow for a better correlation with multifarious definitions and classification systems currently used in the medical literature.

In this study, we did not encounter any persons with a celiac-bi-mesenteric trunk. This was not surprising as it is extremely rare [10, 14], with a global prevalence of only 0.02%. The “classic” complete CMT was present in 0.45% of unselected persons in our study sample, and this was statistically similar to the global prevalence (0.82%).

The most common incomplete CMT variant in our sample was the hepato-mesenteric trunk (1.8%), but it was significantly less prevalent in our sample than was seen across the globe (3.24%; *P*=0.0352). Due to the multifarious existing classifications, comparisons were challenging because some authors reporting on the hepato-mesenteric trunk attempted to distinguish between variants with the CHA arising from the SMA versus a replaced or accessory HA arising from the common trunk [10–12, 19, 28, 31]. Others attempted to define the hepato-mesenteric trunk according to the terminal HA branches arising from the common trunk [9, 21]. One publication even attempted to make an unclear distinction between “a combination of splenogastric and hepato-mesenteric trunk” separate from “splenogastric trunk with CHA arising from the SMA,” both categories being reported individually [10]. In our study, we did not attempt to distinguish between persons with CHA and accessory or replaced hepatic arteries arising from the common trunk because there is clinical significance that once any one of these vessels arises from the common origin. The clinical significance of these variations is discussed below.

The only other incomplete CMT variant we encountered was the hepato-spleno-mesenteric variant in 0.15% of unselected persons, and this was statistically similar to the 0.45% global incidence of this variant. There are existing reports on the hepato-spleno-mesenteric trunk in the medical literature [10, 11, 14, 18, 20, 25–27, 36, 37, 47, 48, 67, 70–72], although there is some variation in descriptions. For example, Loukas et al. [73] described a 74-year-old woman with an “anomalous splenic artery which arose as a branch of the mesenteric artery and gave rise to the common hepatic artery.” Although they did not use nomenclature, a detailed review of the anatomic description and published photographs reveal that this was actually a hepato-spleno-mesenteric trunk. Similarly, Hemant et al. [74] described a case in which the “SMA gave hepatosplenic trunk as its first branch,” and then, the “hepatosplenic trunk divided into the splenic artery and a branch to the common hepatic artery.” Published photographs suggest this is also a hepato-spleno-mesenteric trunk.

## 5. Clinical Significance

Some authors have noted that a CMT can be associated with other arterial variants [1, 9, 18, 27, 42, 46, 69], morphologic anomalies [11, 56, 63], and/or clinical sequelae [62, 63]. None of the patients in our study had clinical sequelae attributable to the presence of a CMT, and they were all incidentally discovered during imaging for other diagnoses. Nevertheless, awareness of this anatomic variation carries great clinical significance when it comes to invasive surgical or interventional procedures.

Interventional radiologists are often required to perform selective angiography of the aortic branches to identify a source of haemorrhage in patients with gastrointestinal bleeding, diagnostic angiography in trauma patients with solid visceral injuries, infusion of transarterial hepatic chemotherapy, angioembolization for pancreatic pseudoaneurysms, and solid organ injuries. In these cases, the presence of the CMT increases technical complexity and impacts procedural planning. These variations are also important in surgical practice as they herald technical difficulty at operation and require modification of operative procedures. The variation in CMT origin and course of the arterial branches increases the risk of iatrogenic arterial injury during pancreaticoduodenectomy, hepatectomy, and gastrectomy. For example, a CHA arising from a CMT often takes an aberrant course posterolateral to the pancreatic neck, which puts it at great risk of injury during a pancreaticoduodenectomy, leading to intraoperative haemorrhage, hepatic ischaemia, biliary strictures, anastomotic leaks, or death.

In addition, some oncologic operations require the division of arteries at their origin in order to achieve a proper nodal harvest. For example, the surgical oncologist is required to divide the left gastric artery at its origin during a gastrectomy or distal oesophagectomy [10, 26, 66]. These variations also increase the complexity of transplant surgery as it may increase the risk of graft failure [10, 66, 75] and also require modification of operative techniques. For example, Guglielmo et al. [66] reported a modification of their transplant techniques requiring harvesting an aortic patch of the common trunk in organ procurement for liver transplantation.

In some cases, the presence of the CMT may be beneficial. For example, during suprarenal aneurysmorrhaphy, the vascular surgeon would be able to harvest and re-implant a single aortic patch to the prosthetic graft to maintain visceral perfusion instead of performing multiple re-implantations of CT and mesenteric arteries. On the other hand, the lower origin of the CMT or CBMT may impact the landing zone for endovascular stent prostheses. Bordei et al. [46] reported that 42% of persons with a CMT had a low origin from the aorta at the lower body of the L1 or L1/2 intervertebral disk.

### 5.1. Study Limitations

We considered whether the low CMT incidence in our study sample was due to human error or misinterpretation. However, all scans were performed on high-resolution multislice scanners with conventional arterial phase protocols and were independently reviewed by three radiologists with specialist interests in vascular anatomy. Therefore, we believe that these data are representative of the variations in this study sample.

## 6. Conclusion

In Caribbean populations, 99.3% of unselected persons have conventional upper abdominal aortic branch anatomy. There was no difference in the prevalence of the classic CMT in the Caribbean compared to the global prevalence (0.45% vs. 0.82%, respectively). However, the hepato-mesenteric trunk (incomplete CMT variant) was significantly less prevalent in the Caribbean (1.8% vs. 3.24%, respectively). Healthcare professionals performing hepatobiliary interventions must be aware of these differences in order to minimize morbidity during their interventions.

## Figures and Tables

**Table 1:** Anatomic variants of the ventral branches of the abdominal aorta in 665 persons.

Anatomic variant	Description: arterial origin from the abdominal aorta	*N* (%)
**Celiacomesenteric trunk**	Arteries belonging to the celiac trunk and mesenteric artery territories have a common origin from the abdominal aorta	16 (2.4%)

(i) **Complete**	A common origin for all arterial branches from CT and mesenteric artery territories	
(a) Celiacomesenteric	A common origin for all arterial branches from CT and SMA territories only	3 (0.55%)
(b) Celiac-bi-mesenteric	A common origin for all arterial branches of the CT, SMA, and IMA territories	0

(ii) **Incomplete**	A common origin for some arterial branches from CT and mesenteric artery territories	
(a) Gastro-mesenteric	A common origin for the left gastric artery and SMA	0
(b) Spleno-mesenteric	A common origin for the splenic artery and SMA	0
(b) Hepato-mesenteric	A common origin for the hepatic artery and SMA	12 (1.8%)
(c) Hepato-spleno-mesenteric	A common origin for the hepatic artery, splenic artery, and SMA	1 (0.15%)
(d) Gastro-spleno-mesenteric	A common origin for the left gastric, splenic, and SMA	0
(e) Hepato-gastro-mesenteric	A common origin for the hepatic artery, left gastric, and SMA	0

CT = celiac trunk; SMA = superior mesenteric artery; IMA = inferior mesenteric artery.

**Table 2:** Analysis of population-based studies evaluating variants of the celiacomesenteric trunk.

Country	Author	Method	Study sample	Conventional branches	CMT variants	Complete CMT		Incomplete CMT					
						Celiac-bi-mesenteric	“Classic” CMT	G-M	S-M	H-M	H–S-M	G-S-M	H-G-M
France	Piquand et al., 1910 [15]	Cadaver	50	41	1	0	1	0	0	0	0	0	0
France	Rio branco, 1912 [16]	Cadaver	50	45	1	0	1	0	0	0	0	0	0
USA	Lipshutz et al., 1917 [1]	Cadaver	83	62	2	0	2	0	0	0	0	0	0
USA	Eaton et al., 1917 [17]	Cadaver	206	186	1	0	1	0	0	0	0	0	0
Japan	Adachi et al., 1928 [18]	Cadaver	252	218	10^E^	0	6	0	0	1	3	0	0
USA	Michels et al., 1955 [9]	Cadaver	200	110	32^E^	0	0	0	1	31^E,5^	0	0	0
USA	Nelson et al., 1988 [19]	Cadaver	50	12	13^E^	0	3	0	0	10	0	0	0
Japan	Shoumura et al., 1991 [20]	Cadaver	450	408	12^E^	0	5	0	0	5	2	0	0
U.S.A.	Hiatt J. et al., 1994 [21]	Transplant operations	1,000	757	15^E^	0	0	0	0	15	0	0	0
Australia	Jones et al., 2001 [22]	Transplant dissection	180	164	30^E^	0	3	0	0	27	0	0	0
Japan	Nakamura et al., 2003 [23]	Cadaver	250	NR	3^E^	0	0	0	0	3	0	0	0
Italy	Ferrari et al., 2007 [24]	Imaging (CT)	60	34	6^E^	0	1	0	0	5	0	0	0
Italy	Ieezi et al., 2008 [25]	Imaging (CT)	524	378	2^E^	0	0	0	NR	NR	2	0	0
Japan	Chen et al., 2009 [26]	Cadaver	974	875	49^E^	0	7	0	0	33	7	2	0
South Korea	Song et al., 2010 [27]	Imaging (CT)	5002	4,457	232^E^	0	53	1	9	132	34	3	0
Russia	Egorov et al., 2010 [28]	Imaging (CT)	350	197	58^E^	0	0	0	0	58^E,4^	0	0	0
Poland	Kornafel et al., 2010 [29]	Imaging (CT)	201	192	4^E^	0	3	0	0	1	0	0	0
Japan	Natsume et al., 2011 [30]	Imaging (CT)	175	159	9^E^	0	1	0	0	5	1	1	1
Spain	Villa et al., 2012 [31]	Imaging (CT)	100	85	15^E^	0	1	0	0	14^E,2^	0	0	0
Japan	Miyaki et al., 2012 [32]	Cadaver	378	NR	8^E^	0	2	0	0	6	0	0	0
Romania	Matusz et al., 2012 [33]	Review	10,750	9,751	73	0	73	NR^1^	NR^1^	NR^1^	NR^1^	NR^1^	NR^1^
Greece	Panagouli et al., 2013 [10]	Systematic review	12,196	10,906	446^E^	1	93	0	0	297/9829^E,7^	49	6	0
China	Mu et al., 2103 [34]	Imaging (CT)	102	60	1	0	1	0	0	0	0	0	0
Serbia	Ognjanovic et al., 2014 [35]	Imaging (CT)	150	117	19^E^	0	4	0	0	15	0	0	0
China	Wang et al., 2014 [36]	Imaging (CT)	1500	1,347	135^E^	0	23	0	18	67	26	1	0
Romania	Iacob et al., 2014 [37]	Imaging (CT)	5442	4,942	30^E^	0	23	0	0	NR	7		0
China	Huang et al., 2015 [38]	Imaging (CT)	238	220	10^E^	0	2	0	2	6	0	0	0
India	Sharma et al., 2015 [39]	Imaging (CT)	80	66	3	0	3	0	0	0	0	0	0
India	Babu et al., 2015 [11]	Imaging (CT)	682	548	19^E^	0	3	1	0	12^E,3^	3	0	0
Poland	Torres et al., 2015 [40]	Imaging (CT)	1569	1455	72^E^	0	8	0	0	64	0	0	0
Japan	Yuasa et al., 2016 [41]	Imaging (CT)	279	253	8^E^	0	3	0	1	3	1	0	0
Egypt	Osman et al., 2016 [42]	Imaging (CT)	1000	905	142^E^	0	6	0	0	136	0	0	0
Iran	Farghadani et al., 2016 [12]	Imaging (CT)	607	308	72^E^	0	4	0	2	66^E,6^	0	0	0
Turkey	Alsaner et al., 2017 [43]	Imaging (CT)	1000	890	4^E^	0	1	NR	NR	NR	3	0	0
Pakistan	Khan et al., 2017 [44]	Imaging (CT)	160	139	2^E^	0	1	NR	NR	NR	1	0	0
Turkey	Caliskan et al., 2018 [45]	Imaging (CT)	174	157	10^E^	0	3	0	1	5	1	0	0
Mexico	Pinal-Garcia et al., 2018 [13]	Cadaver	140	61	4^E^	0	0	0	1	3	0	0	0
Romania	Bordei et al., 2019 [46]	Imaging (CT)	2220	NR	12	0	12	NR	NR	NR	NR	NR	NR
China	Tang et al., 2019 [47]	Imaging (CT)	5580	5031	475^E^	0	96	0	67	248	57	4	3
China	Mao et al., 2019 [48]	Imaging (CT)	2500	2243	225^E^	0	85	0	27	112	1	0	0
India	Ramadevi et al., 2020 [49]	Cadaver	25	21	1	1	0	0	0	0	0	0	0
Czech Republic	Whitley et al., 2020 [14]	Meta-analysis	17,391	15,639	620^E^	0	125	4	30	378	78	5	0
**Global prevalence**	**Global**		**74,320**	**63,439/71,472 (88.8%)**	**2,886/74,320 (3.88%)**	**2/74,320 (0.003%)**	**659/74,320 (0.89%)**	**6/60,190 (0.01%)**	**159/59,666 (0.27%)**	**1,758/54,224 (3.24%)**	**276/61,350 (0.45%)**	**22/61,350 (0.04%)**	**4/61,350 (0.007%)**
**Caribbean**	Cawich et al.	Imaging (CT)	665	649 (97.6%)	16/665 (2.41%)	0	3 (0.45%)	0	0	12 (1.8%)	1 (0.15%)	0	0
**P value**	Caribbean vs. global				0.054	—	0.2985	—	—	**0.0352**	0.3798	—	-

*E* = extrapolated from raw data and/or published images. 1 = these authors reported an “incomplete CMT” in 63 (0.59%) cases but did not clearly define the meaning of this term. 2 = CHA from SMA (3) + accessory RHA from SMA (2) + replaced RHA from SMA (9). 3 = individual cases included CHA from SMA and LGA/SA from CT (9) + CHA from SMA and LGA/SA arising directly from the aorta (1) + replaced RHA from SMA (2). 4 = individual cases included CHA from SMA (9) + replaced RHA from SMA (49). 5 = individual cases included CHA from SMA (9) + replaced RHA from SMA (22). 6 = individual cases included CHA from SMA (8) + RHA from SMA (58). 7 = individual cases included splenogastric and hepato-mesenteric trunk (186) + splenogastric with CHA arising from SMA (111)

## Data Availability

Data will be made available from the corresponding author upon reasonable request.
